# Expression of MIR155HG, LOC283856, KIAA0125, and LOC100190986 as potential prognostic and predictive biomarkers for breast cancer

**DOI:** 10.1590/1414-431X2025e14805

**Published:** 2026-02-06

**Authors:** A.C. Pavanelli, F.R.R. Mangone, G.P. de Jesus, M.A. Nagai

**Affiliations:** 1 Laboratório de Genética Molecular, Centro de Investigação Translacional em Oncologia, Instituto do Câncer do Estado de São Paulo, Faculdade de Medicina, Universidade de São Paulo, São Paulo, SP, Brasil; 2 Centro de Estudos e Tecnologias Convergentes para Oncologia de Precisão, Universidade de São Paulo, São Paulo, SP, Brasil; 3 Disciplina de Oncologia, Departamento de Radiologia e Oncologia, Faculdade de Medicina, Universidade de São Paulo, São Paulo, SP, Brasil

**Keywords:** Breast cancer, lncRNAs, Biomarker, Prognosis, MIR155HG

## Abstract

Accumulating evidence has pointed out that the altered expression of long non-coding RNAs (lncRNAs) is involved in the physiopathology of breast cancer (BC). However, the role of lncRNAs in BC progression remains poorly understood. Here, we evaluated cDNA microarray data from a previous study from our group to investigate the effects of SPARC expression on the transcriptome of MCF7 cells before and after treatment with docetaxel. We analyzed our gene expression data to identify differentially expressed long non-coding RNAs (DELncRNAs). In combination with *in silico* analysis, we selected a group of DELncRNAs with potential prognostic and predictive value for BC patients with tumors of different intrinsic subtypes. Overall, we identified 260 DELncRNAs comparing MCF7 cells with different expressions of SPARC after docetaxel treatment. Nine DELncRNAs (LOC646762, FLJ13224, CASC2, LOC100130691, MGC12916, LOC100190986, LOC283856, KIAA0125, and MIR155HG) showed significant associations with BC survival on the KM Plotter platform. Of these 9 DELncRNAs, MIR155HG, LOC283856, LOC100190986, and KIAA0125 were significantly correlated with recurrence-free survival and overall survival rates of BC patients, suggesting they could help predict the outcome of BC patients as prognostic factors. Moreover, *in silico* analysis showed that these DELncRNAs were able to predict BC patients' responses to different treatment protocols.

## Introduction

Breast cancer (BC) ranks highest in incidence and mortality among cancers affecting women worldwide and is considered an important public health problem. In 2020, 2.3 million women were diagnosed with BC, and 685,000 deaths were reported worldwide (1). BC has a diverse etiology and is classified into four main molecular subtypes: Luminal A, Luminal B, HER2-positive, and triple-negative (TN). Luminal tumors are characterized by the presence of estrogen receptors (ER) and progesterone receptors (PR), along with the absence of the HER-2 oncogene. Luminal B tumors may express PR at either high or low levels, and HER-2 can also be detected in some cases. Due to their hormone receptor-positive status, these tumors can benefit from endocrine therapies, such as selective estrogen receptor modulators (SERMs), selective estrogen receptor degraders (SERDs), and aromatase inhibitors (AIs). Patients with HER-2-positive breast tumors may respond positively to treatment with monoclonal antibodies. In contrast, triple-negative tumors lack expression of the ER, PR, and HER-2 receptors. The conventional treatment approaches for these tumors typically includes chemotherapy and surgery. BC is a complex disease with variability in its molecular, pathological, and clinical course, and its response to treatment (2). Despite advances in the molecular classification of BC, new biomarkers are needed for prognosis and prediction of patients' responses to different therapeutic strategies.

Evidence has indicated that long non-coding RNAs (lncRNAs) play critical roles in the pathophysiology of BC (3). As reported in the 2023 consensus, lncRNAs constitute a group of transcripts composed of 500 nucleotides or more that do not have the potential to encode proteins (4). They can be located in the genome in sense, antisense, bidirectional, intronic, and intergenic states (5). LncRNAs can regulate gene expression primarily through interaction with different proteins at single or complex interactions, including epigenetic, transcriptional, post-transcriptional, translational, and post-translational levels (5). Aberrant expressions of several lncRNAs have been reported to be associated with BC (6,7). In addition, different lncRNAs are differentially expressed and harbor prognostic and predictive value in breast cancer (8,9). Although the research on lncRNAs has increased significantly in recent years, the number and function of the lncRNAs associated with BC have not been elucidated and require further studies.

Docetaxel is a widely used and effective anti-microtubule agent in the treatment of BC (10). However, the mechanisms associated with chemosensitivity or resistance to docetaxel are not yet clearly defined. The SPARC (secreted protein acidic, and rich in cysteine) is a 42 KDa matricellular protein that acts at the interface between the cell surface and the extracellular matrix (ECM) and can directly or indirectly modulate the action of several growth factors involved in cell adhesion, cell migration, tissue remodeling, angiogenesis, embryogenesis, and tumorigenesis (11). SPARC expression has been associated with sensitivity to chemotherapeutic drugs *in vivo* and *in vitro* (12) and to taxanes (13). Furthermore, the GEparSepto-GBG6 trial has reported that BC patients, mainly of the triple negative subtype, with increased SPARC expression had a significant increase in response to nab-paclitaxel (14).

Using cDNA microarray analysis to identify potential biomarkers involved with docetaxel chemosensitivity, we evaluated the effects of SPARC expression on the transcriptome of MCF7 cells before and after treatment with docetaxel [data not published (ACP and MAN)]. Here, we explored our gene expression data generated using cDNA microarrays to identify differentially expressed lncRNAs (DELncRNAs), and in combination with *in silico* analysis, we selected a group of DELncRNAs with a potential prognostic and predictive value for patients with different BC subtypes.

## Material and Methods

### Cell line, plasmid transfection, and docetaxel treatment

MCF-7 cell line was purchased from the ATCC (American Type Culture Collection, USA) and maintained in RPMI medium supplemented with 10% fetal bovine serum (GIBCO, USA) containing antibiotic-antimycotic (100 U/mL penicillin, 100 μg/mL streptomycin, and 0.25 μg/mL of fungizone) (GIBCO) in an incubator at 37°C under an atmosphere of 5% CO_2_. MCF-7 cells were stably transfected with the empty expression vector pCMV6-Neo (empty vector) or containing the complete cDNA of the SPARC gene (pCMV6-SPARC) (Origene, USA). For the transfection assay, 1×10^5^ MCF7 cells were distributed in 6-well plates, and after achieving the necessary confluence (60-70%), they were transfected using the Turbofectin 8.0 reagent (Origene), following the manufacturer's instructions. Clones transfected with the vectors pCMV6-Neo (control) and pCMV6-XL6-SPARC were selected with 3,000 μg/mL of the geneticin antibiotic to establish a lineage with permanent expression of the SPARC gene. Then, the selected clones were maintained in a lower concentration of geneticin (800 μg/mL). After selection, the stable clones were screened for SPARC expression by immunocytochemistry and western blotting. MCF7*pc*Neo and MCF7*pc*SPARC cells were treated with the chemotherapy drug docetaxel (Taxotere; Aventis Pharmaceutics Inc., USA) dissolved in absolute ethanol (0.6 mM stock solution). All experiments were performed in triplicate. The concentration was 100 nM docetaxel, and control cells were treated with the same final amount of absolute ethanol (less than 0.01%) for 24 h.

### cDNA microarray analysis

For microarray assays, total RNA was extracted from control MCF7*pc*Neo and MCF7*pc*SPARC cells treated with 100 nM docetaxel for 24 h, using the guanidine isothiocyanate and phenol-chloroform methodology, previously described by Chomczynski and Sacchi (15). RNA concentration was determined by the NanoDrop ND-1000 spectrophotometer (Thermo Scientific, USA), and the absorbances at 260 and 280 nm were also evaluated, in which the ideal 260/280 ratio was between 1.9 and 2.0. The samples were also evaluated for integrity using RNA Nano Chips on an Agilent 2100 Bioanalyzer device (USA), using samples that presented a RIN>8. After this step, 200 ng of RNA purified by the RNasey Mini Kit (Qiagen, Brazil), according to the manufacturer's instructions, was used to carry out the amplification step. The purified RNAs from the control and treated samples and the RNA used as a reference (Universal Human Reference RNA - Agilent) with the addition of control RNA (Spike-in) were amplified using the Low Input RNA Linear Amplification kit according to the manufacturer's instructions (GE Healthcare Bio-Sciences Corp., USA). Subsequently, the T7 RNA polymerase enzyme was added with the fluorochromes cyanine 5 (Cy-5) as an experimental sample and cyanine 3 (Cy-3) as a reference sample for amplification steps of complementary anti-sense RNAs (cRNA). After this step, the cRNAs were purified using the RNasey Mini Kit (Qiagen), and 825 ng of labeled cRNA was hybridized with Human GE 4X44K V2 slides (Agilent Technologies) for 17 h at 65°C in a hybridization oven for microarrays. Slides were digitized by Agilent Microarrays Scanner, and data quantification and quality control were performed using Agilent Feature Extraction (FE) Software 12.1 (https://www.agilent.com/cs/library/usermanuals/public/GEN-MAN-G4460-90055.pdf). The extracted data was imported into the Agilent Gene Spring 12.5 - GX Analysis Program. The present data were deposited in NCBI's Gene Expression Omnibus (16) and will be available through GEO Series accession number GSE241892.

### Analysis in public databases

We compared the expression profile of MCF7pcSPARC cells *vs* MCF7pcNeo cells after treatment with docetaxel to identify the differentially expressed genes (DEGs) using a cut-off criteria of P<0.05 and |log_2_FC|≥2.0. Among the DEGs, we filtered the DELncRNAs, excluding all coding RNAs and pseudogenes from the list. Analyses in public databases were performed manually for each DELncRNA. The UALCAN platform (http://ualcan.path.uab.edu/), which contains the Cancer Genome Atlas (TCGA) expression data (17) was used to compare the expression profile between normal and tumor tissue and between different subtypes of breast cancer. To select the main DELncRNAs in relation to their impact on patient survival - both overall survival (OS) and recurrence-free survival (RFS) - we used the Kaplan-Meier Plotter platform (http://kmplot.com), which contains a large number of clinical and gene expression data from BC patients (18). Jetset probes were used, and the best cutoff was selected with a survival time of 120 months. We also evaluated the potential of DELncRNAs in predicting response to chemotherapy treatment with taxanes - HER2-directed targeted therapy - indicated for BC patients with tumors presenting amplification or overexpression of HER2, and endocrine therapy, indicated for BC patients with ER and PR positive tumors, using the ROC plotter tool platform (http://www.rocplot.org/) (18). The reference numbers of the selected jetset probes are indicated at the top of each KM Plotter and ROC Plotter charts. The selected DELncRNAs with the most significant prognostic and predictive potential for response to treatment were used in conjunction with SPARC to build networks using the NetworkAnalyst software (https://www.networkanalyst.ca/) (19).

## Results

When comparing the expression profile of MCF-7 cells expressing SPARC (MCF7*pc*SPARC) and MCF-7 cells without SPARC expression (MCF7*pc*Neo) after treatment with docetaxel, we observed a total of 260 DELncRNAs, where 107 were up-regulated and 153 were down-regulated. All DELncRNAs identified were searched in the UALCAN, KM Plotter, and ROC Plotter platforms. Seventeen DELncRNAs identified as down-regulated and 13 as up-regulated were found in all three platforms. Next, the potential biomarker candidates were selected based on their prognostic value for BC using the KM Plotter platform. In this verification phase, nine DELncRNAs (LOC646762, FLJ13224, CASC2, LOC100130691, MIR155HG, MGC12916, LOC100190986, LOC283856, and KIAA0125) showed good correlation with BC prognosis. The study workflow chart is shown in Supplementary Figure S1. Finally, the following DELncRNAs were selected as having high potential to be candidate biomarkers for BC: MIR155HG, LOC283856, and KIAA0125.

**Figure 1:**
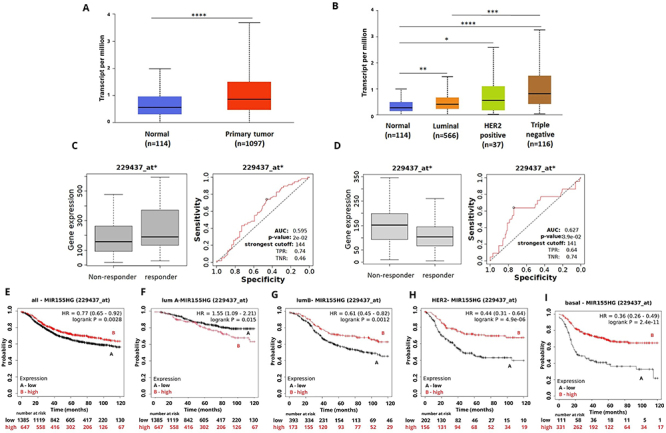
MIR155HG expression and its association with treatment response and survival of breast cancer (BC) patients. **A**, MIR155HG expression in BC samples compared to normal breast tissue. **B**, MIR155HG expression pattern among the BC intrinsic subtypes. **C**, MIR155HG expression for pathological complete response (PCR) groups in the responder and non-responder BC patients under any anti-HER2 therapy. **D**, MIR155HG lncRNA expression for relapse-free survival (RFS) groups in the responder and non-responder BC patients under any endocrine therapy. **E**-**I**, Kaplan-Meier curves for RFS of BC patients for (**E**) all subtypes or each intrinsic subtype: (**F**) luminal A, (**G**) luminal B, (**H**) HER2, and (**I**) basal grouped as high or low expression of MIR155HG using the best cut-off value and the JetSet best probe set (229437_at). Data are reported as medians and interquartile range. *P<0.05; **P<0.001; ***P<0.0001; ****P<0.00001 (Mann-Whitney test).

**Figure 2:**
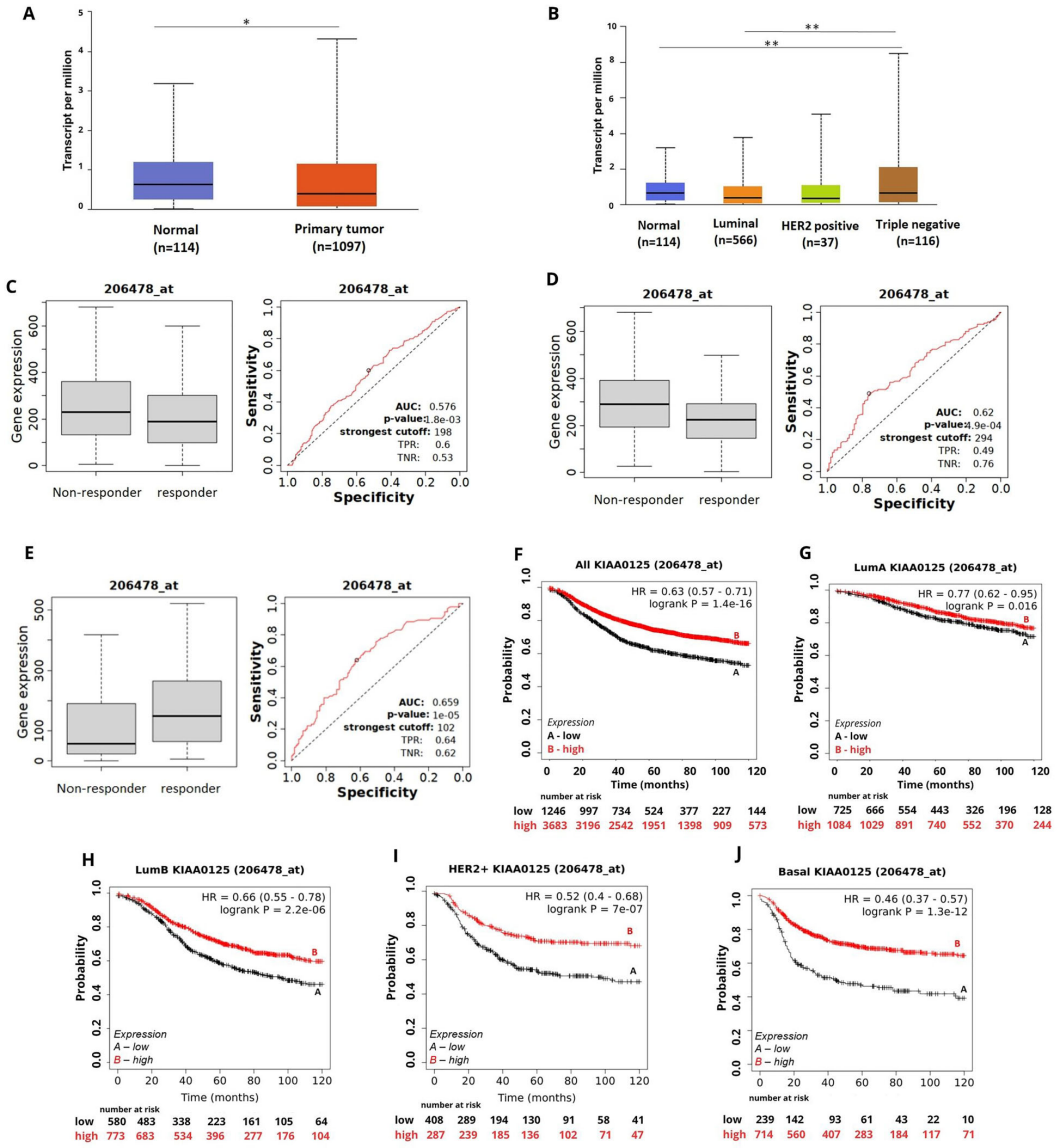
KIAA0125 expression and its association with treatment response and survival of breast cancer (BC) patients. **A**, KIAA0125 expression in BC samples compared to normal breast tissue. **B**, KIAA0125 expression pattern among BC intrinsic subtypes. **C**, KIAA0125 expression for recurrence-free survival (RFS) groups in the responder and non-responder BC patients under any chemotherapy. **D**, KIAA0125 expression for RFS groups in the responder and non-responder BC patients under taxanes treatment. **E**, KIAA0125 expression for RPC groups in the responder and non-responder BC patients under anti-HER2 therapy. **F**-**J**, Kaplan-Meier curves for relapse-free survival (RFS) of BC patients for (**F**) all subtypes or for each intrinsic subtype as (**G**) luminal A, (**H**) luminal B, (**I**) HER2, and (**J**) basal grouped as high or low expression of KIAA0125 using the best cut-off value and the JetSet best probe set (206478_at). Data are reported as medians and interquartile range. *P<0.05; **P<0.001 (Mann-Whitney test).

**Figure 3:**
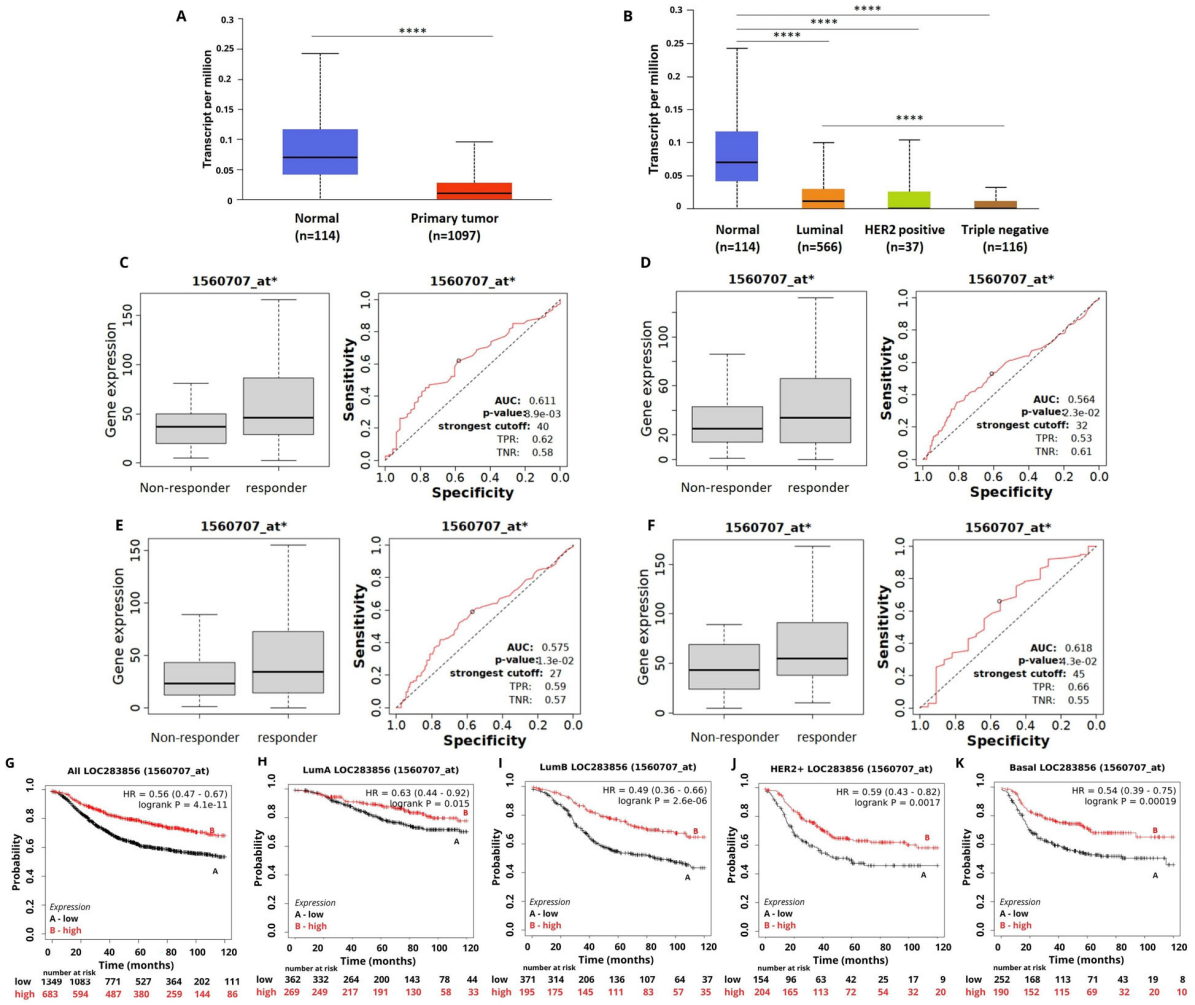
LOC283856 expression and its association with treatment response and survival of breast cancer (BC) patients. **A**, LOC283856 expression in BC samples compared to normal breast tissue. **B**, LOC283856 expression among different BC intrinsic subtypes. **C**, LOC283856 expression for recurrence-free survival (RFS) groups in the responder and non-responder BC patients under any chemotherapy. **D**, LOC283856 expression for RPC groups in the responder and non-responder BC patients treated with any chemotherapy. **E**, LOC283856 expression for RPC groups in the responder and non-responder BC patients under taxane treatment. **F**, LOC283856 expression for RFS groups in the responder and non-responder BC patients under any endocrine therapy. **G**-**K**, Kaplan-Meier curves for relapse-free survival of BC patients for (**G**) all subtypes or for each intrinsic subtype: (**H**) luminal A, (**I**) luminal B, (**J**) HER2, and (**K**) basal grouped as high or low expression of LOC283856 using the best cut-off value and the JetSet best probe set (1560707_at). Data are reported as medians and interquartile range. ****P<0.0001 (Mann-Whitney test).

**Figure 4:**
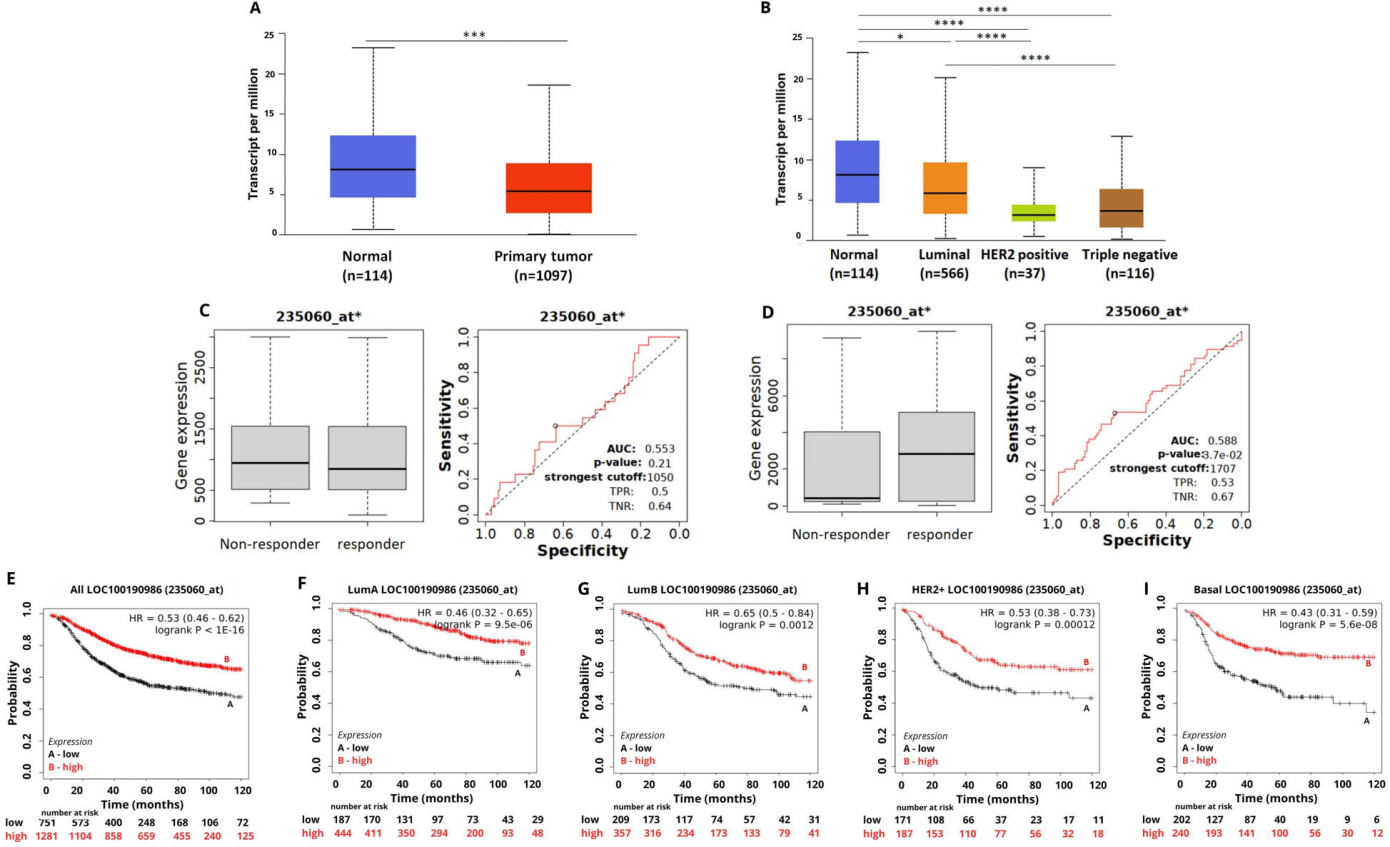
LOC100190986 expression in breast cancer (BC) and association with treatment response and patient survival. **A**, LOC100190986 expression in BC samples compared to normal breast tissue. **B**, LOC100190986 expression pattern among different BC intrinsic subtypes. **C**, LOC100190986 expression for relapse-free survival (RFS) groups in the responder and non-responder BC patients under any endocrine therapy. **D**, LOC100190986 expression for pathological complete response (PCR) groups in the responder and non-responder BC patients under any anti-HER2 therapy. **E**-**I**, Kaplan-Meier curves for RFS of BC patients for (**E**) all subtypes or for each intrinsic subtype: (**F**) luminal A, (**G**) luminal B, (**H**) HER2, and (**I**) basal grouped as high or low expression of LOC100190986 using the best cut-off value and the JetSet best probe set (235060_at). Data are reported as medians and interquartile range. *P<0.05; ***P<0.001 (Mann-Whitney test).

The expression profile of the nine selected DELncRNAs was evaluated in the TCGA database using the UALCAN platform. In the comparison between normal tissue and BC, only the lncRNA MIR155HG showed upregulation in breast tumors in relation to the normal tissue (Figure 1A). The other 8 DELncRNAs - lncRNAs KIAA0125 (Figure 2A), LOC283856 (Figure 3A), LOC100190986 (Figure 4A), LOC646762, CASC2, FLJ13224, LOC100130691, MGC12916 (Supplementary Figure S2) - were downregulated in BC compared to the normal tissue. When comparing the different BC subtypes, MIR155HG (Figure 1B) and KIAA0125 (Figure 2B) transcripts were more expressed in the TN BC subtype, while the LOC283856 transcripts were less expressed in TN BC (Figure 3B), and LOC100190986 (Figure 4B) was more expressed in the luminal subtype. The expression profiles of LOC646762, FLJ13224, CASC2, LOC100130691, and MGC12916 in the different BC subtypes are shown in Supplementary Figure S3.

**Figure 5:**
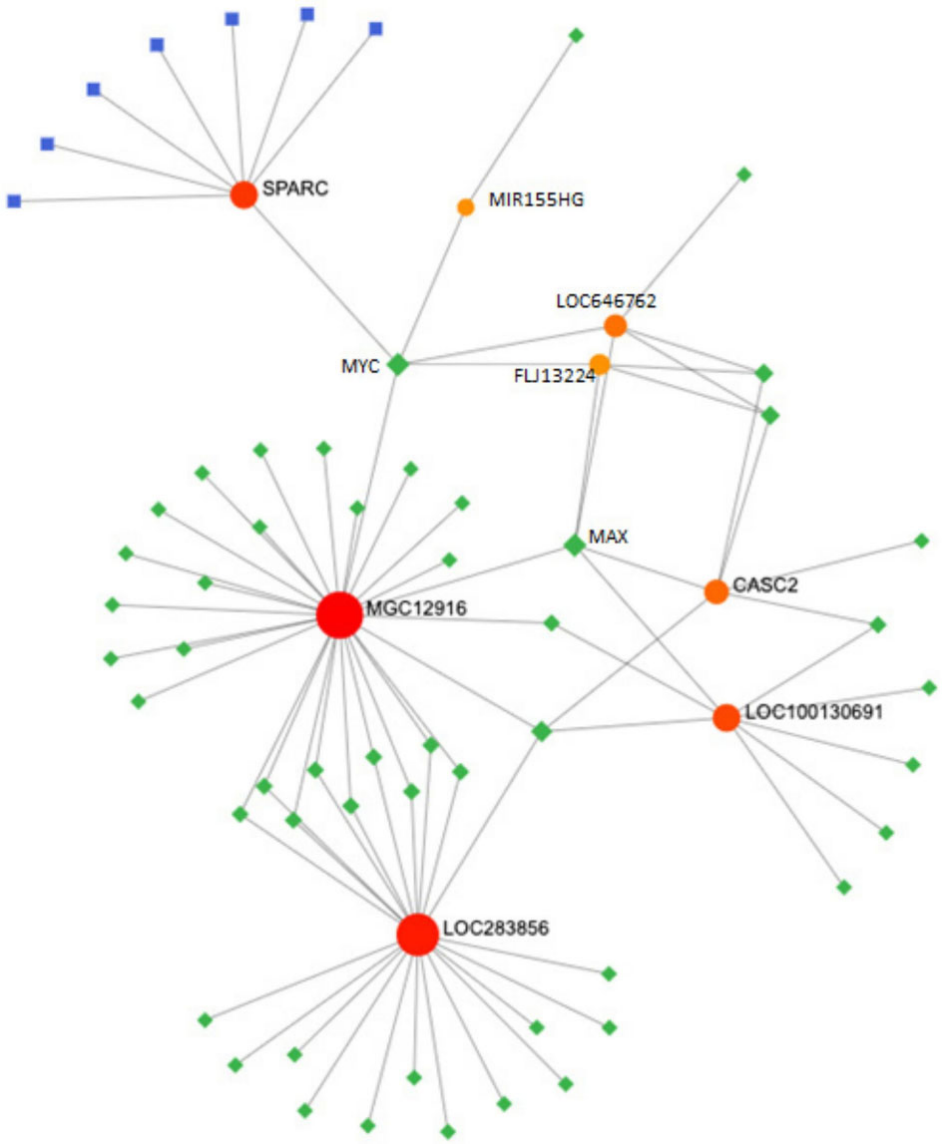
Transcription factor (TF)-miRNA coregulatory network - a global network of TF and lncRNA. TFs are colored in green, the lncRNa are colored in orange, and the mRNA are colored in blue and red (https://www.networkanalyst.ca/NetworkAnalyst/Secure/vis/NetworkView.xhtml).

The potential prognostic value of the selected DELncRNAs was evaluated on the KM Plotter platform. We generated simple Kaplan-Meier estimates without adjusting for confounders. Downregulation of MIR155HG was correlated with reduced rates of RFS in BC patients independent of the molecular subtype (Figure 1E).

Reduced expression of MIR155HG was correlated with shorter RFS and OS in patients with luminal B subtype (P<0.01, Figure 1G), HER2-positive (P<0.0001, Figure 1H), and TN BC (P<0.0001, Figure 1I) tumors, while high expression of MIR155HG was correlated with shorter survival in patients with luminal A subtype tumors (RFS, P=0.015, Figure 1F; OS, P=0.025, Supplementary Figure S4.

Reduced expression of KIAA0125 stratified patients with poor RFS (Figure 2F-J) and OS (Supplementary Figure S5) for patients with the different BC subtypes, except for OS of patients with the luminal A subtype.

LOC283856 downregulation was correlated with poor RFS in patients with all BC subtypes (Figure 3G-K) and short OS for patients with the luminal B BC subtype (Supplementary Figure S6). On the other hand, overexpression of LOC283856 was correlated with short OS for patients with TNBC (Supplementary Figure S6, P=0.028).

Reduced expression of DELncRNA LOC100190986 was correlated with shorter RFS in BC patients independent of the molecular subtype (Figure 4E-I). No statistically significant correlations were observed between the expression of LOC100190986 and OS (Supplementary Figure S7).

The survival curves obtained by the KM Plotter platform for the DELncRNAS LOC646762, FLJ13224, CASC2, MGC12916, and LOC100130691 are shown in Supplementary Figures S8-S12.

We further investigated the potential predictive value of the selected DELncRNAs through the ROC plotter database. Although the AUC values did not clearly discriminate the population, the expression of MIR155HG affected the response to anti-HER2 therapy and endocrine therapy (Figure 1C and D). The expression level of MIR155HG in responders to anti-HER2 therapy was higher than in non-responders (AUC 0.595; P<0.05). However, low expression levels of MIR155HG were found in BC patients who responded to any endocrine therapy (AUC 0.627, P<0.05).

BC patients that responded to any chemotherapy (AUC 0.576, P<0.05) or taxanes (AUC 0.62, P<0.05) showed low levels of KIAA0125 expression and responders to any hormone therapy showed high levels of this DELncRNA (Figure 2C-E).

High expression of LOC283856 predicted the effect of any chemotherapy (AUC 0.611, P<0.05), taxane treatment (AUC 0.575, P<0.05), and hormone therapy (AUC 0.618, P<0.05) (Figure 3C-F).

A high expression level of LOC100190986 predicted a positive response in BC patients to any chemotherapy (AUC 0.63, P<0.05), taxane treatment (AUC 0.601, P<0.05), and anti-HER2 therapy (AUC 0.588, P<0.05) (Figure 4C and D). The above results suggest that the selected DELncRNAs may predict treatment response of BC.

Finally, we searched the selected DELs in the RNAInter (http://www.rnainter.org/) platform and found that the majority were associated with transcription factors (TF). This observation led us to construct a TF-miRNA coregulatory network using the Network Analyst software to gain insight into the potential biological relevance of the selected DELncRNAs. To build the network, we inputted 9 DELncRNAs (LOC646782, FLJ13224, CASC2, LOC100130691, MIR155HG, MGC12916, LOC100190986, LOC283856, and KIAA0125) and SPARC. This analysis revealed the interaction of some differentially expressed lncRNA and SPARC with MYC and MAX proteins (Figure 5). It also revealed seven miRNAs to be key regulators of SPARC (hsa-mir-203, hsa-mir-495, hsa-mir-192, hsa-mir-204, hsa-mir-29a, hsa-mir-29b, and hsa-mir-29c).

## Discussion

A growing number of studies have revealed that lncRNAs are involved in the tumorigenic process (20). In BC, several reports identified abnormal expression of different lncRNAs as potential biomarkers of prognosis and treatment response (3). In this study, we used cDNA microarray profiling comparing MCF-7 cells overexpressing SPARC and MCF-7 cells without SPARC expression after docetaxel treatment, followed by *in silico* analysis to identify DELncRNA as candidates for BC prognosis and treatment response.

Overall, we identified 107 upregulated and 153 downregulated DELncRNAs comparing MCF7 cells with different expressions of SPARC after docetaxel treatment. Subsequently, we accessed the potential prognostic value of the DELncRNAs using the KM Plotter platform. The results identified 9 DELncRNAs with prognostic value, being LOC646762, FLJ13224, CASC2, LOC100130691, MGC12916, LOC100190986, LOC283856, and KIAA0125 downregulated and MIR155HG upregulated in the BC tissues compared with normal tissues in the TCGA dataset. Of these, MIR155HG, LOC283856, LOC100190986, and KIAA0125 were significantly associated with RFS and/or OS rates of BC patients, suggesting they could be useful prognostic factors to predict BC patients’ survival. Moreover, *in silico* analysis showed that these DELncRNAs predicted the response of BC patients to different treatment protocols.

MIR155HG (miR155 host gene) is located on chromosome 21, contains three exons, and was first identified in avian leukosis virus-induced lymphomas (21). The lncRNA MIR155HG is expressed in different tissues and is involved in various physiological and pathological processes (22). Altered expression of MIR155HG has been reported in different types of cancer, including glioblastoma, gastric, ovarian, renal, pancreatic and colorectal cancer, and BC (7,23-26). To date, few studies have evaluated the role of MIR155HG in BC. Our findings are in accordance with a large and comprehensive transcriptomic analysis using microarray libraries from gynecologic tumors, which also found upregulated MIR155HG in BC cells compared to normal breast tissue (27). On the other hand, Ghafouri-Fard et al. (28) evaluated the expression of the lncRNAs ITGB2-AS1, HCP5, and MIR155HG in 82 BC tissues and their adjacent non-cancerous tissues and found no significant differences in the expression of MIR155HG between tumor and non-tumor breast tissues. These conflicting findings might reflect differences in the sample studied by Ghafouri-Fard et al. (28), which were from a single center in Iran, and the validation sample used in our study, which was from TCGA, a multicenter study with a large number of samples. There are no reports evaluating the prognostic or predictive value of MIR155HG expression in BC. Here, we found that MIR155HG downregulation was associated with both RFS and OS of luminal B, Her2-positive, and TN BC patients.

The prognostic and predictive value of MIR155HG has been reported for different types of cancer. In clear cell renal cell carcinoma, MIR155HG's high expression was found to correlate significantly with the OS of the patients (7). MIR155HG overexpression was also found to be associated with poor prognosis in patients with pancreatic cancer (19) and ovarian cancer (29). Furthermore, Peng et al. (30) reported an association between high expression of MIR155HG and better OS in cholangiocarcinoma, lung adenocarcinoma, and melanoma, and a high level of MIR155HG was associated with poorer OS in glioblastoma multiforme, kidney renal clear cell carcinoma, brain lower grade glioma, and uveal melanoma.

Experimental studies in different types of cancer have started to reveal some of the functional roles of MIR155HG in tumorigenesis. In cervical cancer, both *in vivo* and *in vitro*, MIR155HG knockdown inhibits malignant progression by inhibiting proliferation and inducing apoptosis in SiHa and Hela cells and inhibits tumor growth in xenograft, effects that were recovered by SRSF1 overexpression (31). In gastric cancer, MIR155HG overexpression increases the malignant phenotype through cell proliferation, colony-forming ability, cell migration ability, and tumor growth in nude mice (32). These authors also demonstrated that inhibition of NF-κB and STAT3 signaling pathways decreases the effects of MIR155HG overexpression in gastric cancer cells (32). MIR155HG knockdown inhibits proliferation, induces G1/S-phase cell cycle arrest, and increases apoptosis in glioblastoma cells (33). Moreover, MIR155HG overexpression can sponge miR-185 and upregulate ANXA2 expression, which increases the level of MIR155HG expression through STAT3 phosphorylation (34). Furthermore, the knockdown of MIR155HG or ANXA2 suppressed M2 macrophage polarization, proliferation, migration, and invasion in colorectal cancer (CRC) cells and inhibited M2 macrophage polarization and CRC progression in nude mice (26).

Using the ROC Plotter platform, we assessed the potential of MIR155HG expression in chemosensitivity in BC. High expression of MIR155HG was associated with response to anti-HER2 therapy, while low levels of this lncRNA were associated with response to endocrine therapy. There are no data on the role of MIR155HG in drug sensitivity in BC. However, Zhou et al. (26) showed that the knockdown of MIR155HG or ANXA2 increased the oxaliplatin resistance of CRC. In glioma, MIR155HG knockdown leads to increased sensitivity to temozolomide (TMZ) by inhibiting Wnt/β-catenin pathway activation (35). MIR155HG overexpression activates STAT3 and NF-κB signaling pathways, reduces apoptosis, and promotes cisplatin and 5-FU resistance in gastric cancer cells (32). Our findings provide, for the first time, insights into the potential prognostic and predictive value of MIR155HG in BC, which provides the basis for further clinical and functional studies.

In this study, we identified for the first time two new lncRNAs - LOC283856 (GNAO1-DT) and LOC100190986 - not yet associated with cancer. Reduced expression of both LOC283856 and LOC100190986 was associated with RFS for patients with BC of all intrinsic subtypes. Furthermore, BC patients with high expression of LOC283856 were responsive to treatments with any chemotherapy, taxanes, and endocrine therapy. We also found that BC patients with tumors with high levels of LOC100190986 expression were responsive to any chemotherapy, endocrine, or anti-HER2 therapies. There are a few studies associating these lncRNAs with prognosis. In ovarian cancer, LOC100190986 was found to be upregulated and associated with poor prognosis in combination with five other lncRNAs. The functional characterization of these lncRNAs remains a critical challenge to the understanding of their role in BC and their potential as biomarkers.

The lncRNA KIAA0125 (also known as FAM30A; family with sequence similarity 30 member A) is located on chromosome 14q32.33, contains six exons, and is expressed in different human tissues. In the TCGA dataset, we found that the expression of the KIAA0125 gene was significantly downregulated in breast tumors compared to normal tissue samples. Here, to elucidate the potential prognostic value of KIAA0125, we evaluated the effects of this lncRNA expression on survival using the KM Plotter platform. We found that KIAA0125 downregulation was associated with reduced RFS rates for BC patients with tumors of the different intrinsic subtypes and short OS for patients with luminal B, HER2-positive, and TNBC tumors. We found no reports in the literature evaluating the expression pattern or prognostic value of KIAA0125 in BC. Altered expression of KIAA0125 has been reported in other tumors. However, its biological functions and role in tumorigenesis remain unclear. KIAA0125 overexpression has been demonstrated in acute myeloid leukemia (AML) and myelodysplastic syndromes (MDS) (33- [Bibr B34] -35), and is already part of a stemness score composed by 17 genes, which could be useful to predict outcomes in AML patients (36). In MDS patients, KIAA0125 overexpression is associated with poor prognosis and correlated with HOX family gene upregulation (33). AML patients with high expression of KIAA0125 had poor prognosis and were more refractory to chemotherapy treatment (37). In AML patients, the KIAA0125 expression level was also associated with RUNX1 mutation and protein fusion (37). Although further studies are needed, KIAA0125 appears to act as an oncogene in both MDS and AML. On the other hand, our present findings in BC and those of the study by Yang et al. (38) in patients with colorectal cancer, showed that low expression of KIAA0125 was associated with a worse prognosis, and its overexpression in HCT116 and SW480 colon cancer cells inhibits proliferation, migration, and invasion, suggesting a tumor suppressor role for KIAA0125.

Furthermore, to gain insights into the interactions and regulations of the selected DELncRNAs, we constructed a TF-miRNA coregulatory network with the nine DELncRNAs and SPARC. Interestingly, SPARC and six of the DELncRNAs showed interactions with MYC and MAX, suggesting a meaningful relationship with these transcription factors, which requires further investigation. However, *in vitro* and *in vivo* studies, including overexpressing and silencing the target lncRNAs, RNA pull-down, ChIP, and luciferase reporter gene assays, are needed to investigate the possible relationship between those transcription factors and the DELncRNAs.

We conducted an exploratory validation study based on public BC expression data sets, providing new information on the involvement of MIR155HG, LOC283856, KIAA0125, and LOC100190986 lncRNAs as potential new biomarkers for BC prognosis. However, we are aware of the study's main limitations. We only analyzed our cell-based profiling data alongside publicly available datasets; preclinical studies with cell lines and animal models were not performed. Therefore, further clinical validation in different cohorts and experimental studies, both *in vitro* and *in vivo*, is necessary to better understand these lncRNAs' actions in the BC tumorigenic process.

In BC, there are distinct molecular subtypes characterized by broader gene signatures such as that initially proposed by Sørlie et al. (39). However, patients with tumors from the same intrinsic molecular group respond differently to the same therapies. Over the years, there have been significant advancements to unravel these differences, and, in this sense, multigene panels (such as OncoType, MammaPrint, and Prosigna, among others) have arisen to identify predictive subgroups in specific BC populations, thereby guiding therapies (40). Despite substantial progress over the years, there is still an urgent need to identify molecules that can improve the molecular classification of BC intrinsic subtypes necessary to gain insights into the molecular mechanisms that control the development and progression of tumors and to devise more efficient clinical tactics for cancer treatment. Within this complex landscape, lncRNAs have emerged as important molecules in gene regulation in BC through protein interactions, transcriptional and post-transcriptional regulations, and epigenetics, among others. Although the structure and function of the DELncRNAs identified here need to be further explored, our findings provide new information on the potential role of lncRNAs that can contribute to refining research targets that better define prognostic groups for tailored therapies.

## Data Availability

All data generated or analyzed during this study were already deposited in NCBI's Gene Expression Omnibus and will be available through GEO Series accession number GSE241892.

## Supplementary Materials

Supplementary Material Click to view [pdf].
