# Supplementary material for: Expression of MIR155HG, LOC283856, KIAA0125, and LOC100190986 as potential prognostic and predictive biomarkers for breast cancer
Source: Braz J Med Biol Res. 2026 Feb 6;59:e14805. doi: 10.1590/1414-431X2025e14805 (PMC12892257; doi:10.1590/1414-431X2025e14805)

Figure S1. Study design flowchart. DElncRNA: differentially expressed long non-coding RNAs.

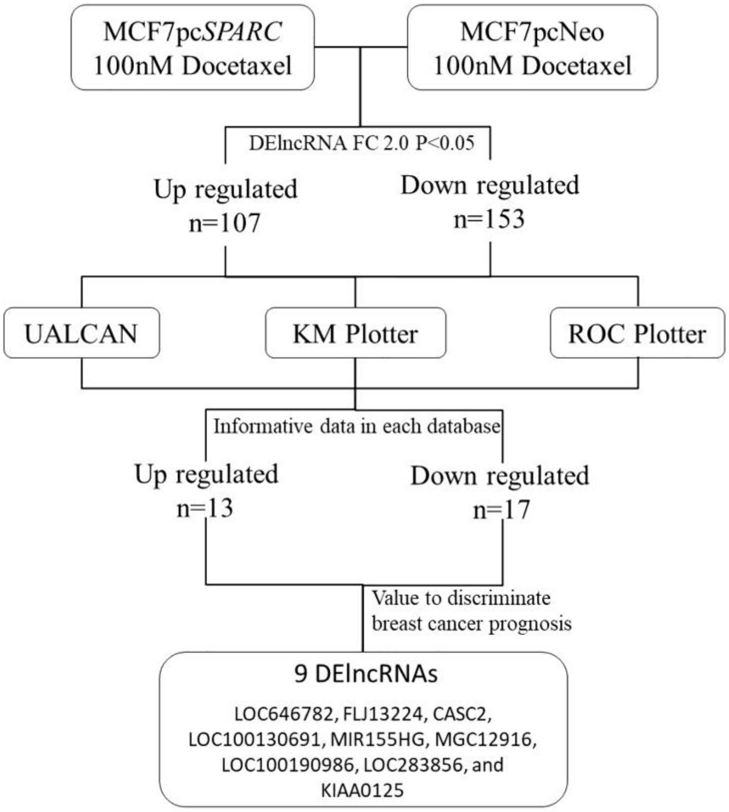

Figure S2. Expression profile of lncRNA in breast cancer (BC) patients. Expression in BC compared to normal breast tissue of (**A**) LOC646762 lncRNA, (**B**) FLJ13224 lncRNA, (**C**) CASC2 lncRNA, (**D**) LOC100130691, and (**E**) MGC12916 lncRNA, using the UALCAN database containing TCGA data. Data are reported as medians and interquartile range. *P<0.05 **P<0.001 ****P<0.0001 (Mann-Whitney test).

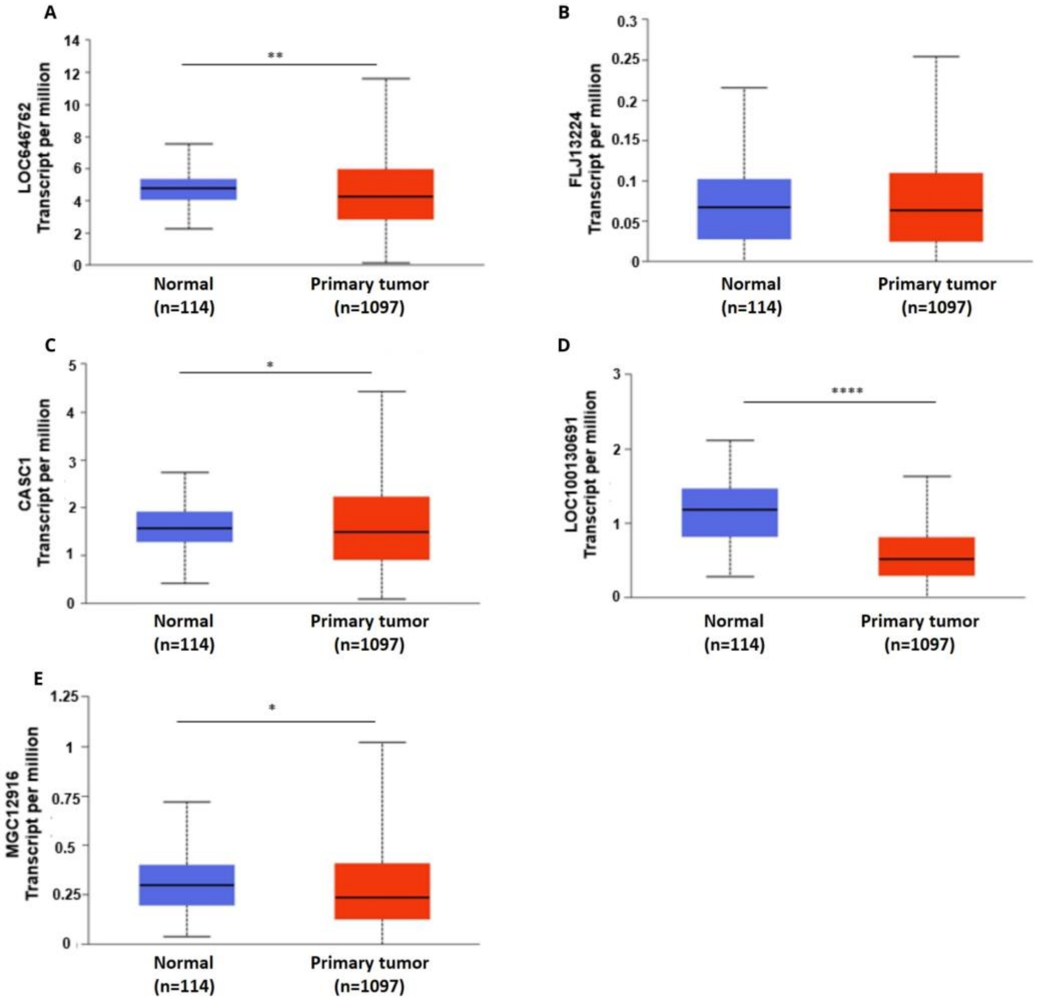

Figure S3. Expression profile of lncRNA in breast cancer patients. Expression in breast cancer different intrinsic subtypes using the UALCAN database containing TCGA data of (**A**) LOC646762 lncRNA, (**B**) FLJ13224 lncRNA, (**C**) CASC2 lncRNA, (**D**) LOC100130691, and (**E**) MGC12916 lncRNA. Data are reported as medians and interquartile range. *P<0.05, **P<0.001, ***P<0.0001, ****P<0.00001 (Mann-Whitney test).

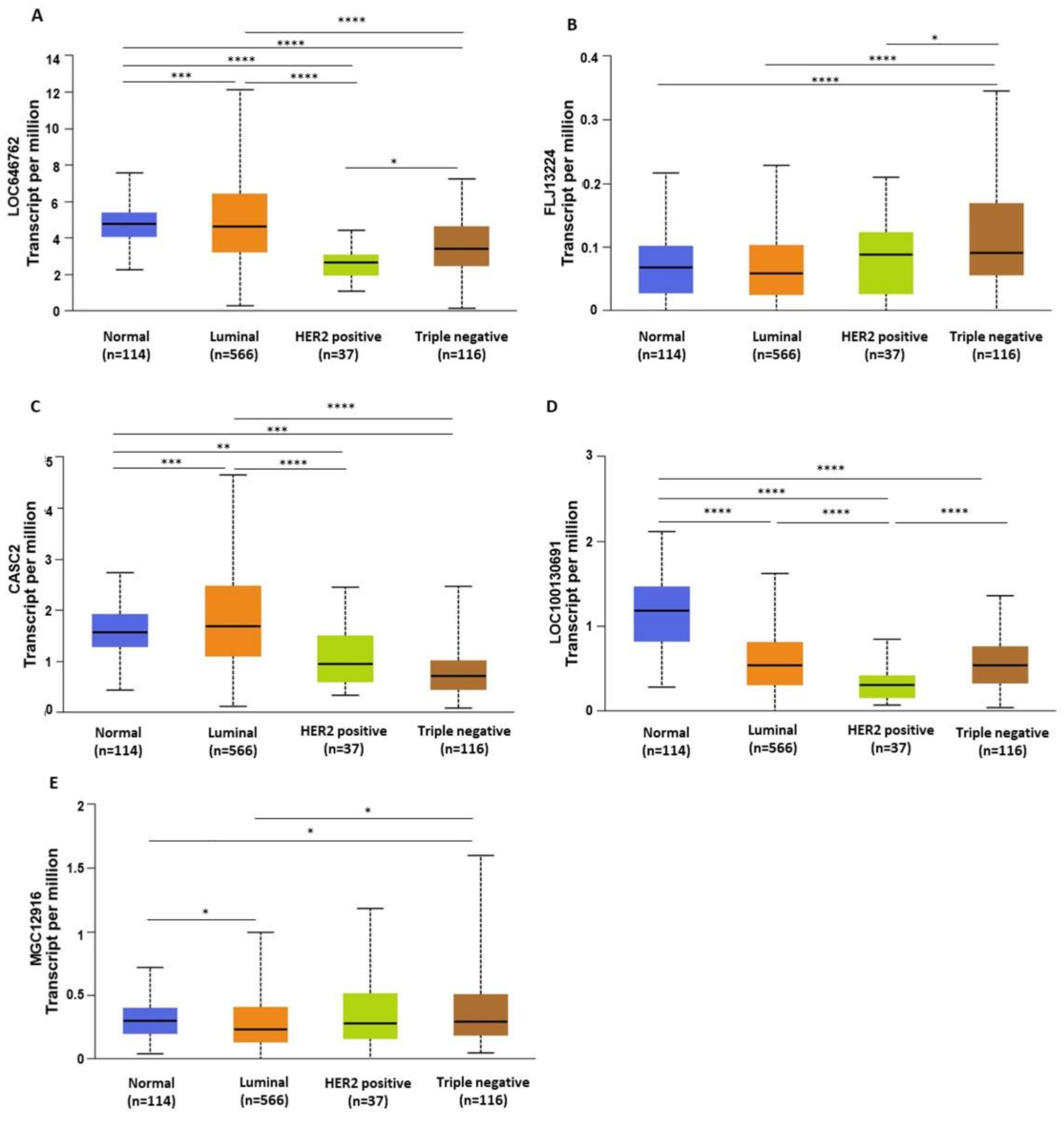

Figure S4. MIR155HG lncRNA expression in breast cancer patients. Kaplan-Meier curves for overall survival of breast cancer patients for (**A**) all subtypes or for each intrinsic subtype: (**B**) luminal A, (**C**) luminal B, (**D**) HER2, and (**E**) basal grouped as high or low expression of MIR155HG according to the best cut-off value using the JetSet best probe set (229437_at) at the KM Plotter online tool.

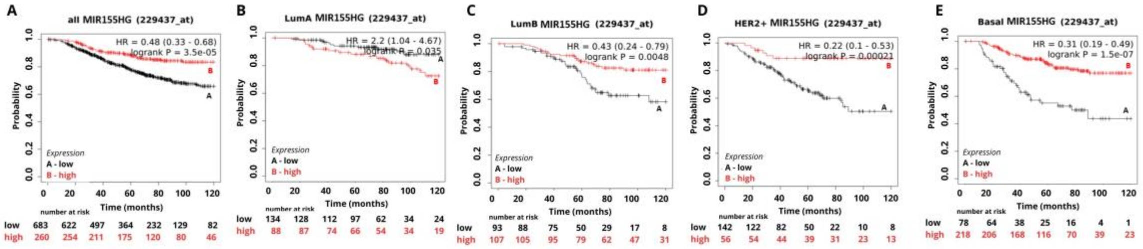

Figure S5. KIAA0125 lncRNA expression in breast cancer patients. Kaplan-Meier curves for overall survival of breast cancer patients for (**A**) all subtypes or for each intrinsic subtype: (**B**) luminal A, (**C**) luminal B, (**D**) HER2, and (**E**) basal grouped as high or low expression of KIAA0125 according to the best cut-off value using the JetSet best probe set (206478_at) at the KM Plotter online tool.

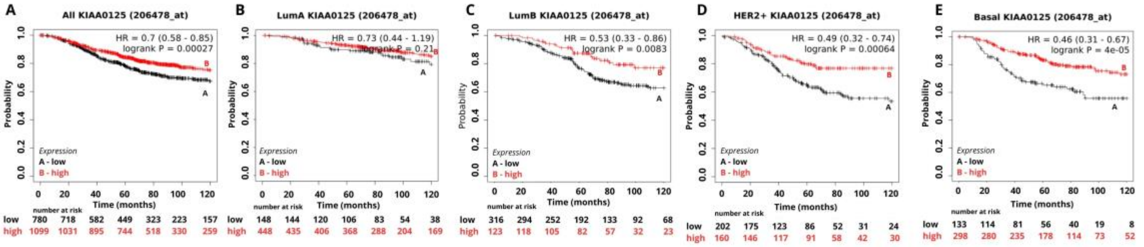

Figure S6. LOC283856 lncRNA expression in breast cancer patients. Kaplan-Meier curves for overall survival of breast cancer patients for (**A**) all subtypes or for each intrinsic subtype: (**B**) luminal A, (**C**) luminal B, (**D**) HER2, and (**E**) basal grouped as high or low expression of LOC283856 according to the best cut-off value using the JetSet best probe set (1560707_at) at the KM Plotter online tool.

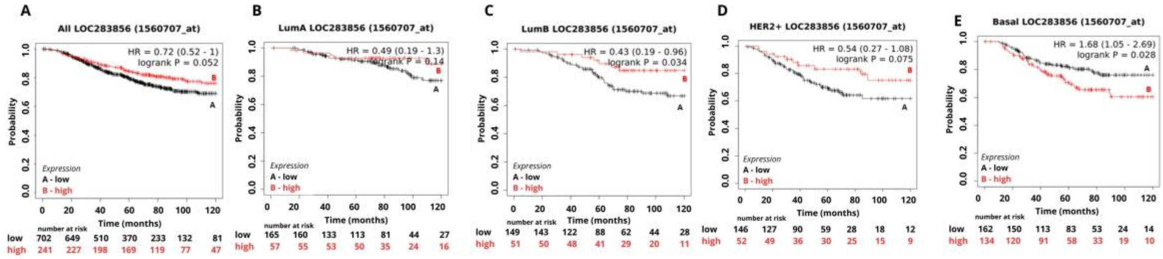

Figure S7. LOC100190986 lncRNA expression in breast cancer patients. Kaplan-Meier curves for overall survival of breast cancer patients for (**A**) all subtypes or for each intrinsic subtype: (**B**) luminal A, (**C**) luminal B, (**D**) HER2, and (**E**) basal grouped as high or low expression of LOC100190986 according to the best cut-off value using the JetSet best probe set (235060_at) at the KM Plotter online tool.

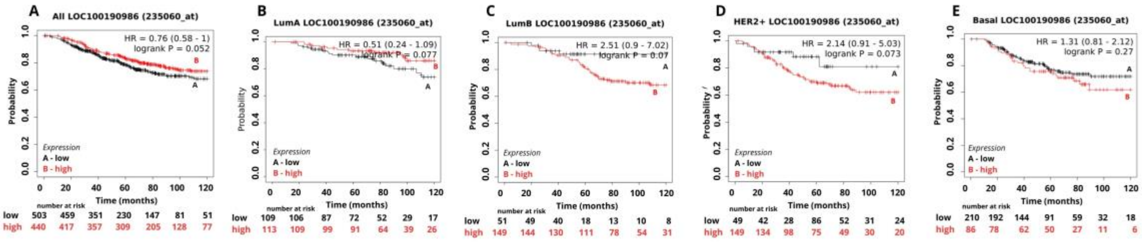

Figure S8. LOC646762 lncRNA expression in breast cancer patients. Kaplan-Meier curves for relapse-free survival of breast cancer patients for (**A**) all subtypes or for each intrinsic subtype: (**B**) luminal A, (**C**) luminal B, (**D**) HER2, and (**E**) basal grouped as high or low expression of LOC646762 according to the best cut-off value using the JetSet best probe set at the KM Plotter online tool. Kaplan-Meier curves for overall survival of breast cancer patients for (**F**) all subtypes or for each intrinsic subtype: (**G**) luminal A, (**H**) luminal B, (**I**) HER2, and (**J**) basal grouped as high or low expression of LOC646762 according to the best cut-off value using the JetSet best probe set (1568597_at) at the KM Plotter online tool.

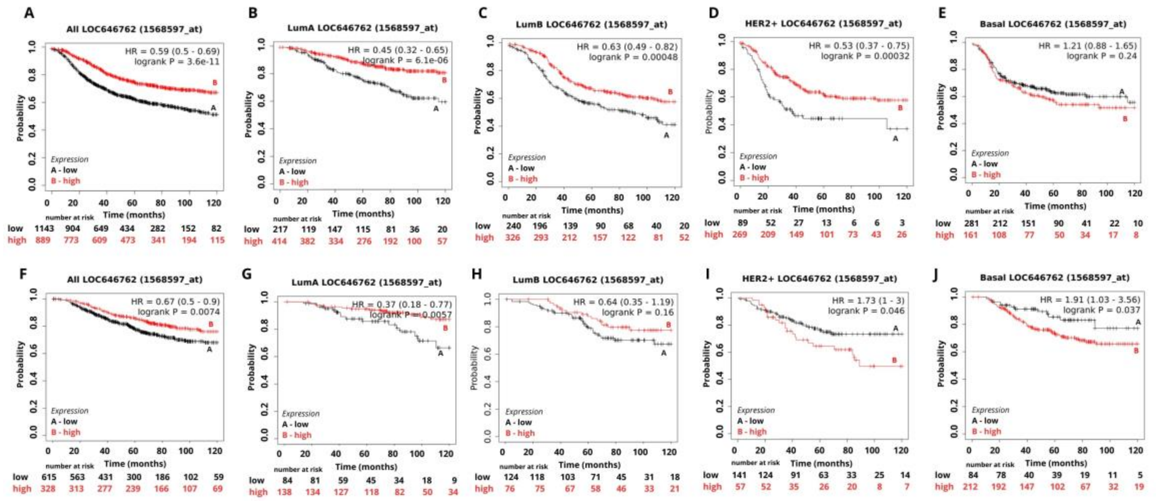

Figure S9. FLJ13224 lncRNA expression in breast cancer patients. Kaplan-Meier curves for relapse-free survival of breast cancer patients for (**A**) all subtypes or for each intrinsic subtype: (**B**) luminal A, (**C**) luminal B, (**D**) HER2, and (**E**) basal grouped as high or low expression of FLJ13224 according to the best cut-off value using the JetSet best probe set at the KM Plotter online tool. Kaplan-Meier curves for overall survival of breast cancer patients for (**F**) all subtypes or for each intrinsic subtype: (**G**) luminal A, (**H**) luminal B, (**I**) HER2, and (**J**) basal grouped as high or low expression of FLJ13224 according to the best cut-off value using the JetSet best probe set (220211_at) at the KM Plotter online tool.

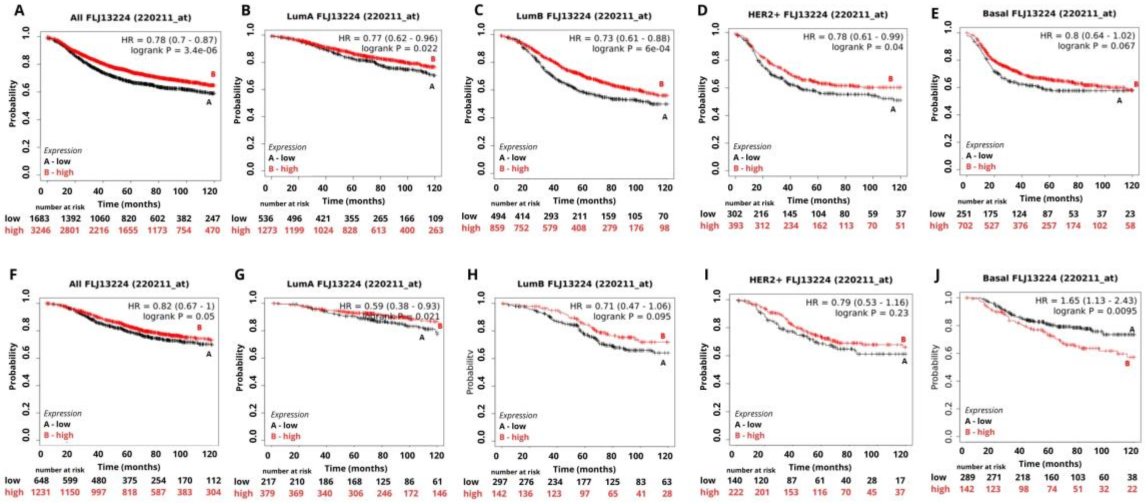

Figure S10. CASC2 lncRNA expression in breast cancer patients. Kaplan-Meier curves for relapse-free survival of breast cancer patients for (**A**) all subtypes or for each intrinsic subtype: (**B**) luminal A, (**C**) luminal B, (**D**) HER2, and (**E**) basal grouped as high or low expression of CASC2 according to the best cut-off value using the JetSet best probe set at the KM Plotter online tool. Kaplan-Meier curves for overall survival of breast cancer patients for (**F**) all subtypes or for each intrinsic subtype: (**G**) luminal A, (**H**) luminal B, (**I**) HER2, and (**J**) basal grouped as high or low expression of CASC2 according to the best cut-off value using the JetSet best probe set (1556630_at) at the KM Plotter online tool.

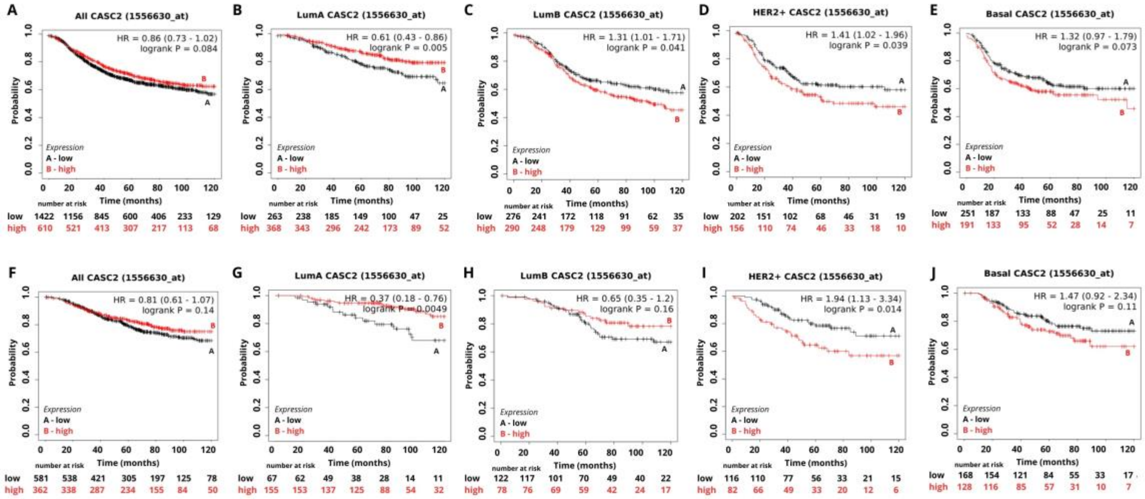

Figure S11. MGC12916 lncRNA expression in breast cancer patients. Kaplan-Meier curves for relapse-free survival of breast cancer patients for (**A**) all subtypes or for each intrinsic subtype: (**B**) luminal A, (**C**) luminal B, (**D**) HER2, and (**E**) basal grouped as high or low expression of MGC12916 according to the best cut-off value using the JetSet best probe set at the KM Plotter online tool. Kaplan-Meier curves for overall survival of breast cancer patients for (**F**) all subtypes or for each intrinsic subtype: (**G**) luminal A, (**H**) luminal B, (**I**) HER2, and (**J**) basal grouped as high or low expression of MGC12916 according to the best cut-off value using the JetSet best probe set (224508_at) at the KM Plotter online tool.

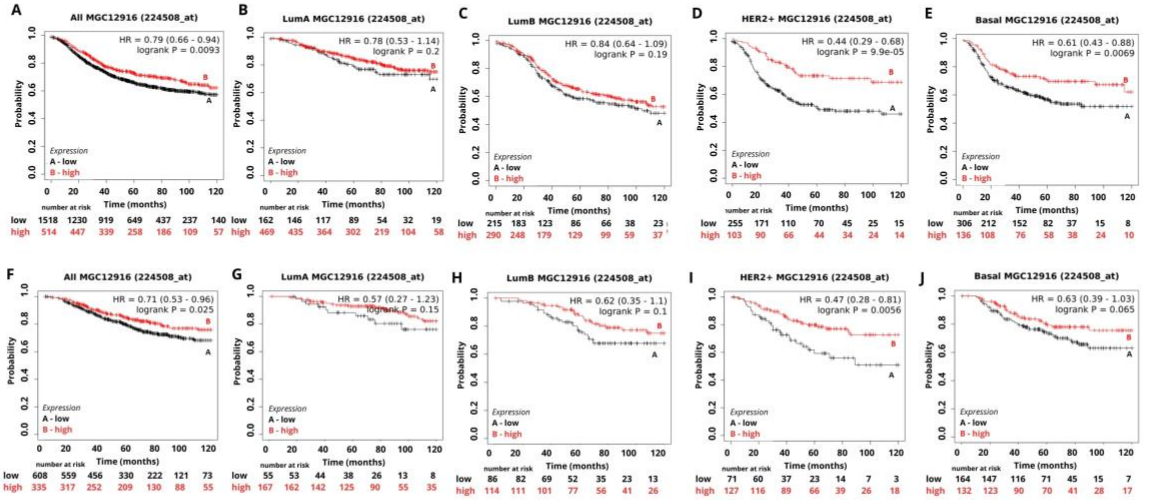

Figure S12. LOC100130691 lncRNA expression in breast cancer patients. Kaplan-Meier curves for relapse-free survival of breast cancer patients for (**A**) all subtypes or for each intrinsic subtype: (**B**) luminal A, (**C**) luminal B, (**D**) HER2, and (**E**) basal grouped as high or low expression of LOC100130691 according to the best cut-off value using the JetSet best probe set at the KM Plotter online tool. Kaplan-Meier curves for overall survival of breast cancer patients for (**F**) all subtypes or for each intrinsic subtype: (**G**) luminal A, (**H**) luminal B, (**I**) HER2, and (**J**) basal grouped as high or low expression of LOC100130691 according to the best cut-off value using the JetSet best probe set (231540_at) at the KM Plotter online tool.

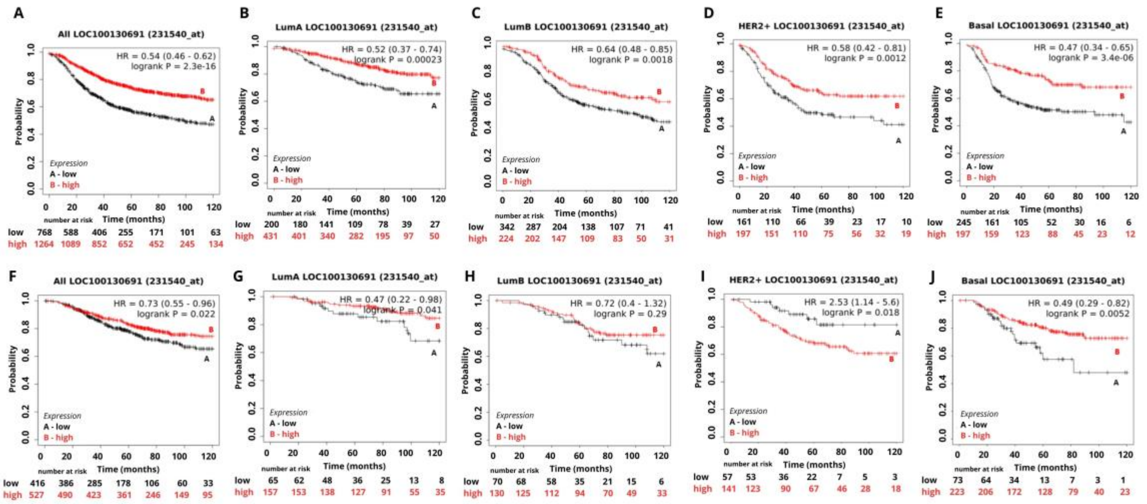

Supplement: Supplementary Material [file 1414-431X-bjmbr-59-e14805-suppl.pdf]
